# Flnc: Machine Learning Improves the Identification of Novel Long Noncoding RNAs from Stand-Alone RNA-Seq Data

**DOI:** 10.3390/ncrna8050070

**Published:** 2022-10-13

**Authors:** Zixiu Li, Peng Zhou, Euijin Kwon, Katherine A. Fitzgerald, Zhiping Weng, Chan Zhou

**Affiliations:** 1 Division of Biostatistics and Health Services Research, Department of Population and Quantitative Health Sciences, University of Massachusetts Chan Medical School, Worcester, MA 01605, USA; 2 Program in Bioinformatics and Integrative Biology, University of Massachusetts Chan Medical School, Worcester, MA 01605, USA; 3 Program in Innate Immunity, Division of Infectious Disease and Immunology, Department of Medicine, University of Massachusetts Chan Medical School, Worcester, MA 01605, USA; 4 The RNA Therapeutics Institute, University of Massachusetts Chan Medical School, Worcester, MA 01605, USA; 5 UMass Cancer Center, University of Massachusetts Chan Medical School, Worcester, MA 01605, USA

**Keywords:** lncRNA, machine learning, RNA-seq, tool, unannotated

## Abstract

Long noncoding RNAs (lncRNAs) play critical regulatory roles in human development and disease. Although there are over 100,000 samples with available RNA sequencing (RNA-seq) data, many lncRNAs have yet to be annotated. The conventional approach to identifying novel lncRNAs from RNA-seq data is to find transcripts without coding potential but this approach has a false discovery rate of 30–75%. Other existing methods either identify only multi-exon lncRNAs, missing single-exon lncRNAs, or require transcriptional initiation profiling data (such as H3K4me3 ChIP-seq data), which is unavailable for many samples with RNA-seq data. Because of these limitations, current methods cannot accurately identify novel lncRNAs from existing RNA-seq data. To address this problem, we have developed software, *Flnc*, to accurately identify both novel and annotated full-length lncRNAs, including single-exon lncRNAs, directly from RNA-seq data without requiring transcriptional initiation profiles. *Flnc* integrates machine learning models built by incorporating four types of features: transcript length, promoter signature, multiple exons, and genomic location. *Flnc* achieves state-of-the-art prediction power with an AUROC score over 0.92. *Flnc* significantly improves the prediction accuracy from less than 50% using the conventional approach to over 85%. *Flnc* is available via GitHub platform.

## 1. Introduction

Only approximately 1% of the human genome and less than 3% of RNA transcripts encode proteins [1]. Long noncoding RNAs (lncRNAs) are a subset of noncoding RNAs longer than 200 nucleotides. Similar to messenger RNAs (mRNAs), most lncRNAs contain 5′ caps and 3′ polyadenylation (polyA) tails [2]. lncRNAs play regulatory roles in numerous biological processes, including human stem cell development and immunity [2,3,4,5,6,7,8,9], and they have been implicated in many diseases, including neurological disorders, cardiovascular, lung, and liver diseases, infectious diseases, and cancer [9,10,11]. lncRNAs are also associated with complex genetic traits, playing important roles in gene regulation by functioning as protein scaffolds, guiding DNA–protein interactions, controlling post-transcriptional regulation, and functioning as cis-regulatory elements at enhancers [12]. However, because evolutionary constraints in noncoding regions are lower than in coding regions, lncRNAs are less conserved and evolve faster than coding genes [13]. Increasingly, studies have shown that lncRNAs exhibit striking disease-specific expression patterns and are potential drivers and modifiers of disease [2,14]. Current databases, such as GENCODE [15], NONCODE [16], and LNCipedia [17], lack annotations for many disease- or cell-type-specific lncRNAs. For example, we previously found that approximately 40% of lncRNAs expressed in human hepatic stellate cells are not yet included in the database [18]. Therefore, it is critical to create a universal tool for the identification of novel lncRNAs which have not been annotated in the current databases, and this tool can be applied across various diseases and developmental processes.

Advances in high-throughput sequencing techniques have produced a large amount of publicly available RNA sequencing data from various tissues, cell lines, and disease models. To date, there are more than 12,700 study series in the NCBI Gene Expression Omnibus (GEO) database with available RNA-seq datasets. This large amount of data offers great opportunities to identify novel lncRNAs expressed in specific biological samples. In addition, the rapid development of computational methods for analyzing sequencing data, including methods for transcript assembly (e.g., Scripture [19], Trinity [20], Cufflink [21], StringTie [22], Strawberry [23], and TransComb [24]) and methods for examining the coding abilities of transcripts (e.g., CPAT [25], LGC [26], PLEK [27], and CPPred [28]), have allowed researchers to generate catalogs of putative lncRNAs—assembled transcripts without protein-coding potential.

However, these methods cannot distinguish between true lncRNAs and false lncRNAs among the putative lncRNAs. Most false lncRNAs are nonfunctional transcribed fragments and transcriptional noise. Unlike the false lncRNAs, true lncRNAs are high-confidence full-length lncRNA transcripts that include transcriptional start sites (TSSs). Additionally, true lncRNAs cannot be distinguished from false lncRNAs based purely on expression levels, because some known functional lncRNAs have low expression levels, such as *VELUCT* [29].

Several approaches have been developed which improve the accuracy of true lncRNAs identification, including examining mammalian conservation, selecting multiple-exon transcripts, or integrating additional transcriptional initiation data to determine TSSs. Each of these suffers from drawbacks. Approaches that identify lncRNA using mammalian conservation [30,31] can fail to detect human-specific lncRNAs, whereas selecting only multiple-exon transcripts to identify multiple-exon lncRNAs have improved accuracy, but will fail to detect single-exon lncRNAs [32]. As over 3000 single-exon lncRNA transcripts have been curated in the GENCODE database, excluding single-exon transcripts in lncRNA identification will overlook many important single-exon lncRNAs. For example, *MALAT1* is a single-exon lncRNA with critical functions in various diseases, ranging from diabetes to cancer [33]. The trimethylation of histone H3 at lysine 4 (H3K4me3) is a chromatin modification known to mark transcription start sites of active genes [34], including lncRNA genes. Therefore, H3K4me3 profiling data (ChIP-seq or CUT-RUN seq data) are commonly used to identify lncRNAs. However, this approach relies on the existence of matched H3K4me3 profiling data and over 96% of the study series with available RNA-seq data in the GEO database lack matched H3K4me3 profiling data. Generating matched H3K4me3 data is often impractical due to limitations on cost, time, and sample availability. This is especially true for patient-derived clinical samples. Therefore, we sought to improve the accuracy of novel lncRNA identification directly from stand-alone (lacking transcription initiation profiles) RNA-seq data.

We developed a machine learning (ML)-based method to distinguish between true and false lncRNAs. Incorporating this new method, we developed *Flnc*, a software program that can directly identify true lncRNAs, including novel and annotated lncRNAs, from stand-alone RNA-seq data. We assessed *Flnc* by comparing the results produced using only RNA-seq data to our previous method [18], which uses RNA-seq and H3K4me3 ChIP-seq data. In five independent test datasets, the *Flnc* pipeline significantly reduced the rate of false positives and achieved over 85% prediction accuracy, significantly outperforming the prediction accuracy of the conventional RNA-seq only method (50%). The *Flnc* pipeline integrates pre-processing, mapping, transcript assembly, evaluation of protein-coding ability, and evaluation of transcripts using our machine-learning algorithm. The *Flnc* pipeline, which is implemented on the user-friendly Singularity platform, has minimal pre-requisites and is easily portable. The *Flnc* can also run with parallelize tasks to optimizes computing resources. The *Flnc* is accessible from https://github.com/CZhouLab/Flnc (accessed on 10 September 2022).

## 2. Results

### 2.1. Generation of a Benchmark Dataset of True and False lncRNAs

**Figure 1:**
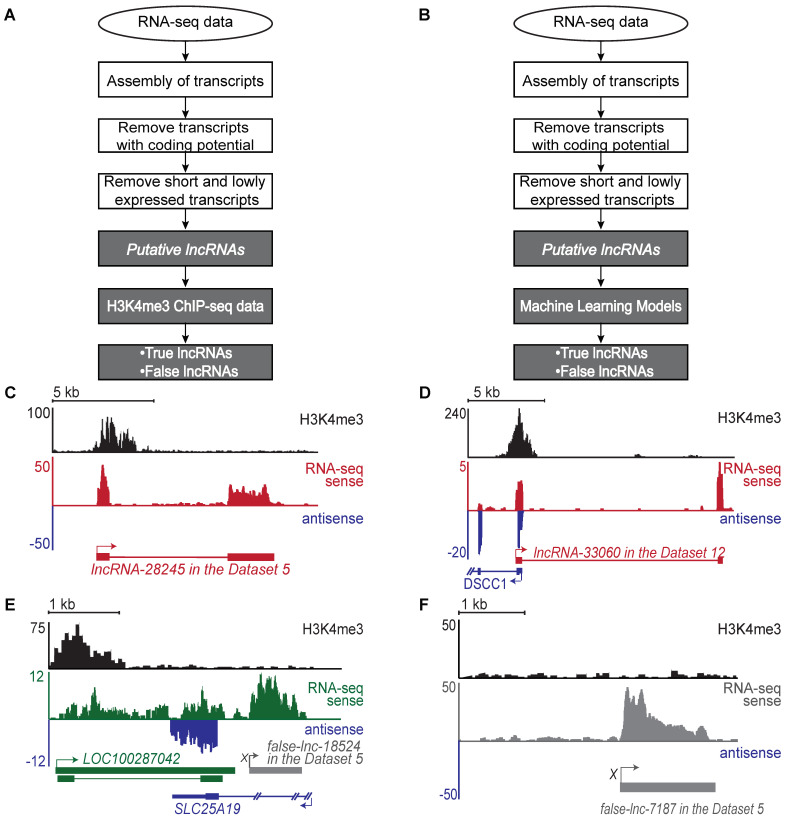

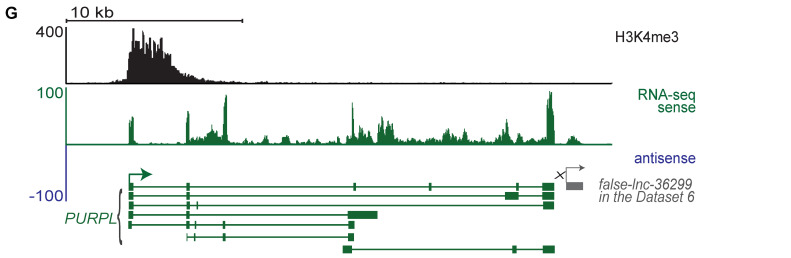
Identification of true and false lncRNAs. (**A**) Flowchart of the standard computational pipeline for identifying true and false lncRNAs (see Section 4 for details). The identified true lncRNAs include both novel and annotated lncRNAs. The standard pipeline requires both RNA-seq data and H3K4me3 ChIP-seq data generated from the same sample. (**B**) Flowchart of *Flnc* pipeline. *Flnc* integrates machine learning models to identify true and false lncRNAs directly from RNA-seq data, without matched H3K4me3 profiles. The *Flnc* pipeline includes two steps: first, *Flnc* identifies putative lncRNAs from RNA-seq data (see Section 4 for details). Then, *Flnc* classifies putative lncRNAs as true or false based on built-in machine learning models. The true lncRNAs predicted by *Flnc* include both novel and annotated lncRNAs. (**C**) A novel true lncRNA detected in Dataset 5. *lncRNA-28245* (red) is located in an intergenic region on chromosome 3. The characteristic H3K4me3 peak identifies it as a true lncRNA. (**D**) A novel true lncRNA identified in Dataset 12. *lncRNA-33060* (red) is located on the sense strand and transcribed divergently from the promoter of the protein-coding *DSCC1* (blue) gene. (**E**) A false lncRNA identified in Dataset 5. False-*lnc-18524.1* (grey) is located downstream of *LOC100287042* (green) and antisense to *SLC25A19* (blue). This false lncRNA is supported by an abundance of RNA-seq reads but lacks H3K4me3 peaks at the 5′ end. The false-lncRNA could be a fragment of an isoform transcript of the *LOC100287042* gene. (**F**) An intergenic false lncRNA identified in Dataset 5. This lncRNA is supported by an abundance of RNA-seq reads but lacks a H3K4me3 peak at the 5′ end. (**G**) A false lncRNA identified in Dataset 6. False-*lnc-36299* (grey) is downstream of the protein-coding gene *PURPL* (green). Compared to *PURPL*, false-*lnc-36299* is expressed at relatively low levels; therefore, it may be the result in RNA polymerase continuing beyond the polyA signal sequences when transcribing *PURPL* (transcriptional noise) [36,37].

To benchmark our new tool, we first updated our previous computational pipeline to use the latest available tools [18,35] (Figure 1A, see Section 4). This pipeline distinguishes between true and false lncRNAs by first identifying putative lncRNAs from raw RNA-seq data, then examining if putative lncRNAs have H3K4me3 peaks near their 5′ ends. When we applied this pipeline to the 46 publicly available datasets with matched RNA-seq and ChIP-seq data, we identified hundreds to thousands of true and false lncRNAs in each individual dataset (Supplementary Figure S1A). Each putative lncRNA found within every dataset was counted as one data point, and this resulted in a total of 244,412 putative lncRNA data points. These putative lncRNAs include 95,265 true lncRNA data points and 149,147 false lncRNA data points. These true and false lncRNA data points constitute our benchmark dataset.

Among all putative lncRNAs in the benchmark dataset, 39% are true lncRNAs, which are supported by both RNA-seq and H3K4me3 ChIP-seq data; these transcripts, with clearly defined transcription start sites (Figure 1C,D), we call true lncRNA. Among these true lncRNAs, 43,941 (over 46%) lncRNAs have not been annotated in the GENCODE database and are called novel lncRNAs.

For the remaining 61% of the putative lncRNAs, we could not locate a transcription start site (Figure 1E–G and Supplementary Figure S1B). These transcripts would be identified as false positive lncRNAs by an RNA-seq only pipeline. Using these criteria, we determined that 30–75% of the putative lncRNAs in each individual dataset were not true lncRNAs (Figure 1E–G and Supplementary Figure S1B). Therefore, without H3K4me3 ChIP-seq data, 30–75% of putative lncRNAs identified from stand-alone RNA-seq can be expected to be false hits.

For each lncRNA track, H3K4me3 peaks mark the site of transcription initiation (black, top). RNA-seq reads supporting true lncRNA transcripts are shown in red (RNA-seq sense). RNA-seq reads supporting annotated genes in the sense strand are shown in green (RNA-seq sense). Antisense transcripts are shown in blue. RNA-seq reads supporting false lncRNAs are shown in gray or in green when these reads continue the RNA-seq reads of supporting protein-coding transcripts. The genomic structure for each gene is shown below the RNA-seq tracks. True lncRNAs are shown in red, false lncRNA are shown in grey, annotated genes in the antisense strand are shown in blue, and annotated genes in the sense strand are shown in green. Boxes represent exons, lines represent introns, and arrows represent the start and direction of transcription. Two annotated genes, *LOC100287042* and *PURPL*, have multiple isoforms. True lncRNAs identified in this study are named with the prefix “lncRNA” followed by the locus number assigned during assembly. False lncRNAs are named with the prefix “false-lnc” followed by the locus number assigned during assembly.

### 2.2. Four Genomic Features Can Be Used to Distinguish True and False lncRNAs

To build a new pipeline (Figure 1B) which can identify lncRNAs from RNA-seq data lacking matched H3K4me3 ChIP-seq data, we first needed to determine which features could distinguish between true and false lncRNA. We hypothesized that a combination of four types of features, transcript length, promoter signature, multiple exons, and genomic location, would provide enough information to distinguish between true and false lncRNAs. Therefore, we used our benchmark dataset to examine these features in true and false lncRNAs.

**Figure 2:**
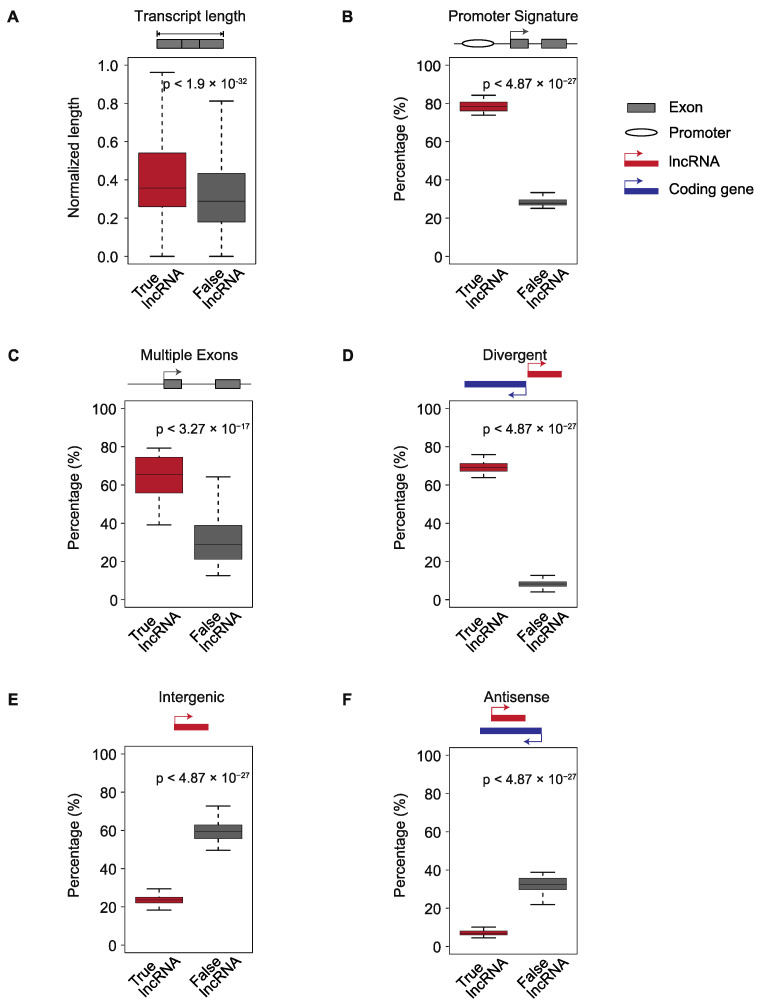
Four features exhibit significant differences between true and false lncRNAs in the 46 benchmark datasets. True lncRNAs can be distinguished from false lncRNAs because they tend to be longer (**A**), to have a predicted upstream promoter signature (**B**), and to be more likely to have multiple exons (**C**). They can also be distinguished by genomic location and are more likely to be divergently transcribed from the promoter of a protein-coding gene (**D**). True lncRNAs are less likely to be in intergenic regions (**E**) or antisense (**F**) to a protein-coding gene. For the graph (**A**), the boxplot represents the scaled transcript lengths of true and false lncRNAs. For the graphs (**B**–**F**), each boxplot represents the percentage of a feature of true and false lncRNAs among the identified putative lncRNAs for each of the 46 benchmark datasets. The *p*-values were calculated by a two-sided Wilcoxon–Mann–Whitney test.

Most false lncRNAs are transcript fragments or transcriptional noise. Therefore, we hypothesized that true lncRNAs would tend to be longer than false lncRNAs. To test this hypothesis, we examined the transcript length of all putative lncRNAs in the benchmark dataset. Read length and sequencing depth differ across various RNA-seq datasets and these factors affect transcript assembly [38,39,40]. To control for these differences, for each of the 46 datasets, we normalized the transcript length of putative lncRNAs to a value between 0 and 1 (see Section 4). After normalization, the transcript lengths of true lncRNAs were significantly longer than those of false lncRNAs (Figure 2A and Supplementary Figure S2A).

Whereas RNA fragments and transcripts resulting from noisy transcription lack promoters and TSSs, true lncRNAs should have upstream promoters. Therefore, we used TSSG software [41] to identify putative promoters upstream of true and false lncRNAs and examined the percentage of true and false lncRNAs with upstream promoter signatures. Almost 80% of true lncRNAs had putative upstream promoters. In contrast, we find this feature in only about 20% of false lncRNAs (Figure 2B and Supplementary Figure S2B).

Because the vast majority of single-exon transcripts result from transcriptional and alignment noise [42,43], we hypothesized that false lncRNAs (transfrags or noise) would have fewer exons than true lncRNAs. To test this hypothesis, we examined the percentage of multiple exon transcripts among true and false lncRNAs. We found that true lncRNAs are significantly more likely to have multiple exons than false lncRNAs (*p*-value < 3.27 × 10^−17^; Figure 2C and Supplementary Figure S2C).

Over 60% of lncRNAs have been shown to be divergently transcribed from the promoter regions of protein-coding genes [18,35]. This suggests that genomic context could be used to distinguish between true and false lncRNAs. We examined the true and false lncRNAs in our benchmark dataset, classifying them into three categories—divergent, antisense, and intergenic—based on their genomic locations. Consistent with previous findings, more than 60% of true lncRNAs were divergent transcripts of protein-coding genes, while less than 20% of false lncRNAs in each of the 46 datasets were divergent transcripts (Figure 2D and Supplementary Figure S2D). Additionally, a small fraction of true lncRNAs originate from intergenic regions or are antisense to coding genes. In contrast, approximately 60% of false lncRNAs are in intergenic regions and 30% are antisense to coding genes (Figure 2E,F and Supplementary Figure S2E,F). Therefore, true and false lncRNAs show significantly different patterns in terms of genomic location.

In conclusion, true and false lncRNAs show significant differences in transcript length, promoter signature, multiple exons, and genomic location. Therefore, we integrated these features into ML models to distinguish between true lncRNAs and other noncoding transcripts.

### 2.3. Training ML Models to Distinguish between True and False lncRNAs

We developed a new computational tool, *Flnc* (Figure 1B) which incorporates the four types of genomic features detailed above into ML models to identify true lncRNA from stand-alone RNA-seq data. We trained seven of the most common ML algorithms using our training set, which comprised 81,420 true and 123,764 false lncRNA that were identified from 41 datasets submitted to the GEO database before 2019. The seven ML algorithms include: logistic regression (LR), k-nearest neighbors (KNN), decision tree (DT), random forest (RF), naïve Bayes (NB), linear kernel support vector machine (SVM), and radial-basis-function (RBF) kernel SVM.

**Figure 3:**
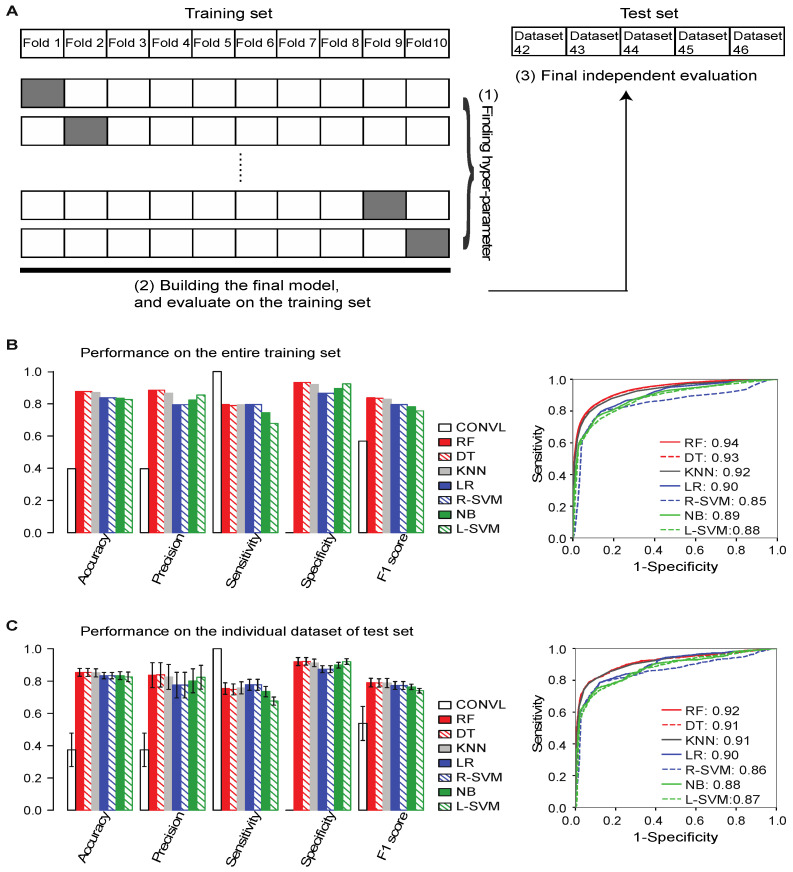
ML model construction and evaluation. (**A**) The overall architecture of *Flnc* training and testing. The best hyperparameters, specifically the hyperparameters yielding the best mean F1 score, were chosen using the 10-fold cross-validation approach as follows. The training set was randomly split into 10 even folds, 9 of which were used in training the models. The 10th fold was used for validation. We repeated this process holding each individual fold out and training with the other 9 folds. From this, we derived 10 models for each hyperparameter setting. For each of the 10 models, we calculated the mean F1 score for each hyperparameter setting. After selecting the best hyperparameter setting, the final model was built using the entire training set and we examined the performance of the final model on the entire training set. Finally, we evaluated the performance of the final model on the test set, which was composed of the five independent datasets generated in 2019 and 2020 (Datasets 42–46). (**B**) The seven final models of *Flnc* outperformed the conventional method (without models) on the entire training set, and (**C**) on the five individual datasets of test set. The left bar graphs of (**B**,**C**) show the performance metrics accuracy, precision, sensitivity, specificity and F1 score. The right graphs (**B**,**C**) show the ROC curve for each model. The AUROC score is shown next to each ML model. The ROC curve of (**C**) is the ROC curve of the seven models on the Dataset 46. Please see Supplementary Figure S3B for the ROC curves of the four additional test datasets (Dataset 42–25). The seven models are ranked by the F1 score from largest to smallest. Abbreviations: CONVL, conventional approach; RF, random forest; DT, decision tree; R-SVM, RBF support vector machine; L-SVM, linear support vector machine; LR: linear regression; NB, naïve Bayes; KNN, and k-nearest neighbors.

To fit and select the best model for each ML algorithm, we first used the 10-fold cross validation approach to train and evaluate each model using all possible combinations of hyperparameters (Figure 3A). We divided the putative lncRNAs in the training set randomly into 10 non-overlapping subsets (folds). For each ML algorithm, we held one of the 10 subsets of putative lncRNAs aside and trained a model using each set of hyperparameter values on the other 9 subsets. We then evaluated the performance of the model on the remaining held-aside data. We repeated the process 10 times, each time holding aside a different subset of data. We considered the hyperparameter values that defined the model with the best mean F1 score—the harmonic mean value of the precision and sensitivity—the optimal model architecture. Next, we trained the optimal model architecture on the entire training set to build the models for *Flnc*.

On the training set, the random forest, decision tree, and KNN models had the best overall prediction performance based on the F1 score and Area Under the Receiver Operating Characteristic (ROC) Curve (AUROC) score (Figure 3B and Supplementary Table S3). The random forest model resulted in F1 = 0.84 and AUROC=0.94 values, the decision tree model resulted in F1 = 0.83 and AUROC = 0.93 values, and the KNN model resulted in F1 = 0.82 and AUROC = 0.92 values. Compared to the other models, these three models also have better accuracy, precision, and specificity. In contrast, the linear SVM was the worst performing of all models with respect to the F1 score, sensitivity, and accuracy.

### 2.4. Flnc Identifies True lncRNAs with up to 87% Prediction Precision

After training the models, we evaluated *Flnc*’s ability to predict true lncRNAs from stand-alone RNA-seq data by testing *Flnc* on a test set composed of five independent datasets that were released to the GEO database after 2019 (Figure 3C). These five datasets were generated from multiple biological samples, including the MOLM-13 human myeloid leukemia cell line, the HUDEP-2 erythroid cell line, Jurkat leukemia cells, and the H1299 non-small cell lung cancer cell line. We evaluated the performance of *Flnc* both on the entire test set (Supplementary Figure S3A and Supplementary Table S3) and on the five individual datasets within the test set (Figure 3C and Supplementary Figure S3B). Consistent with the training set results, the random forest, decision tree, and KNN models have the best overall prediction abilities, as indicated by the F1 and AUROC scores (Figure 3C and Supplementary Figure S3B), although the seven models achieve 72–87% consistency in lncRNA prediction (Supplementary Figure S3C). The three best models achieve 93–96% consistency in lncRNA prediction (Supplementary Figure S3D) with the accuracy of 0.85 or greater, and precision of 0.83 or more. As with the training set, based on the F1 score and accuracy, the linear SVM and naïve Bayes models had the worst performance. Variations in performance between datasets were small (Figure 3C and Supplementary Table S3) with standard deviations in F1 and AUROC scores of less than 0.03 less than 0.02, respectively (Supplementary Table S3). Furthermore, an ensemble approach, which consists of combining the results from all seven models such that a lncRNA is considered true if all seven models predict it, can further improve prediction precision from 83% to 87% and specificity from 92% to 95% at the cost of reducing the sensitivity (Supplementary Figure S3E).

### 2.5. Many lncRNAs Identified by Flnc Are Novel and Are Supported by H3K4me3 Profiles

**Figure 4:**
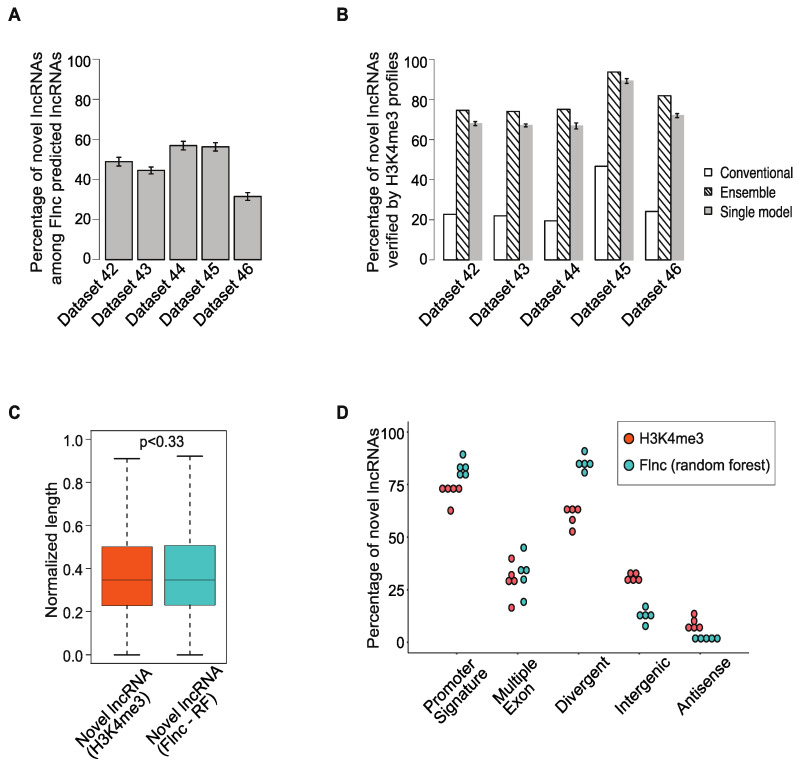
Most novel lncRNAs predicted by *Flnc* are supported by H3K4me3 profiles. (**A**) A large proportion of true lncRNAs predicted by *Flnc* are novel lncRNAs in the five individual test datasets. (**B**) The percentage of novel lncRNAs predicted by the conventional method without models and *Flnc* methods are verified by H3K3me3 profiles. Most novel lncRNAs predicted by the conventional method cannot be verified by H3K4me3 profiles, whereas most of the novel lncRNAs predicted by *Flnc* methods (ensemble approach or each of the seven single models) can be verified by H3K4me3 profiles. The error bars of the single model (gray) bars represent the standard deviations for the results of the seven ML models. (**C**) The novel lncRNAs predicted by *Flnc* (with random forest model) show similar normalized transcript length distribution as the novel lncRNAs determined by H3K4me3 ChIP-seq data. (**D**) The novel lncRNAs predicted by *Flnc* (with the random forest model) includes significantly more divergent transcripts and transcripts with promoter signatures than the novel lncRNAs determined by H3K4me3 ChIP-seq data, whereas multiple exon features exhibit similar percentage between these two groups of novel lncRNAs. Each dot represents one of the five independent datasets in the test set.

Because true lncRNAs include both novel and annotated lncRNAs, we examined the novel lncRNAs among the true lncRNAs predicted by *Flnc* in the five independent test datasets. We found that up to 60% of true lncRNAs predicted by *Flnc* have not yet been annotated in the GENCODE database (Figure 4A). To determine if these novel lncRNAs are true lncRNAs, we examined H3K4me3 ChIP-seq data to determine if these lncRNAs have TSSs; we found that 65–90% of these novel lncRNA are supported by H3K4me3 ChIP-seq data (Figure 4B). Indeed, a novel lncRNA identified by *Flnc* is almost twice as likely to be confirmed by H3K4me3 than one identified using conventional RNA-seq only methods (Figure 4B). Taking the ensemble approach improved the chance of being confirmed even further to between 74% and 94% (Figure 4B).

Next, we examined the genomic features of novel lncRNAs predicted by *Flnc* in the five independent test datasets. Because the random forest model had the best F1 and AUROC scores, we compared the genomic features of novel lncRNAs predicted with the random forest model with those of novel lncRNAs identified by the H3K4me3 ChIP-seq data (Figure 4C,D). The novel lncRNAs predicted by *Flnc* and those identified by H3K4me3 profiles were similar in terms of transcript length (Figure 4C) and multiple exons (Figure 4D), but the novel lncRNAs predicted by *Flnc* were more likely to have promoter signatures and to be divergent transcripts (Figure 4D). We observed similar trends among all the true lncRNAs predicted by Flnc and those determined by H3K4me3 profiles (Supplementary Figure S4A,B).

Additionally, we note that 16–40% of novel lncRNAs predicted by *Flnc* have multiple-exon (Figure 4D) across the five independent test datasets. This means that 60–74% of novel lncRNAs predicted by *Flnc* are single-exon lncRNAs. Similarly, among all the true lncRNAs predicted by *Flnc*, 37–42% of true lncRNAs are single-exon lncRNAs (Supplementary Figure S4D). This indicates that *Flnc* will not miss the single-exon lncRNAs with potential functions.

### 2.6. Flnc Predicts True lncRNAs in Multiple Types of RNA-Seq Samples

**Figure 5:**
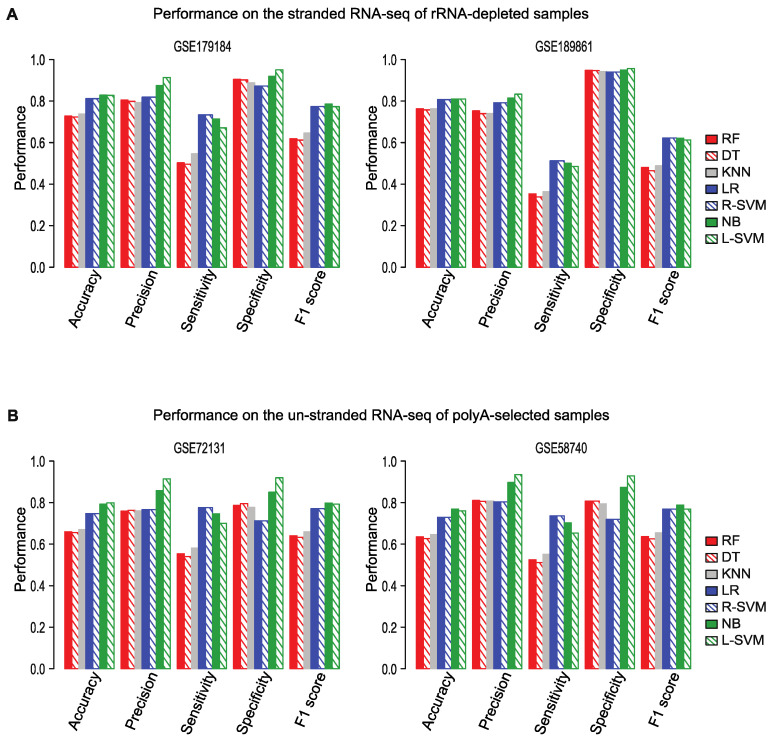
*Flnc* accurately identifies true lncRNAs from rRNA-depleted and unstranded RNA-seq datasets. (**A**) Performance of *Flnc* on two stranded RNA-seq datasets generated from ribosomal RNA (rRNA)-depleted samples (GSE179184 on the left, GSE189861 on the right). (**B**) Performance of *Flnc* on two un-stranded polyA-selected RNA-seq datasets (GSE72131 on the left, GSE58740 on the right).

For both the training and test data, we used stranded RNA-seq data generated from polyA-selected RNA. However, many RNA-seq datasets are generated not from poly-A RNA, but from ribosomal-RNA (rRNA)-depleted RNA and some are sequenced without strand information. To evaluate the performance of *Flnc* in other types of RNA-seq data, we tested *Flnc* on two stranded RNA-seq datasets that were generated using rRNA depletion (Figure 5A) and on two unstranded polyA RNA-seq datasets (Figure 5B).

On both stranded rRNA-depleted RNA-seq and unstranded polyA-selected RNA-seq data, four models—naïve Bayes, linear SVM, RBF SVM, and logistic regression—had similar F1 scores and based on the F1 scores, these four models performed better than other three models (Figure 5). On these types of data, the linear SVM model achieved the best prediction precision and accuracy; the naïve Bayes model ranks as the second best by the precision metric. Although the random forest, decision tree, and KNN models ranked as among the best models when run on polyA-selected RNA-seq data, their performance on rRNA-depleted RNA-seq data ranked as the worst by the metrics of accuracy, precision, sensitivity, and F1 score (Figure 5).

### 2.7. Divergent Transcription Is the Most Important Feature for Predicting True lncRNAs

**Figure 6:**
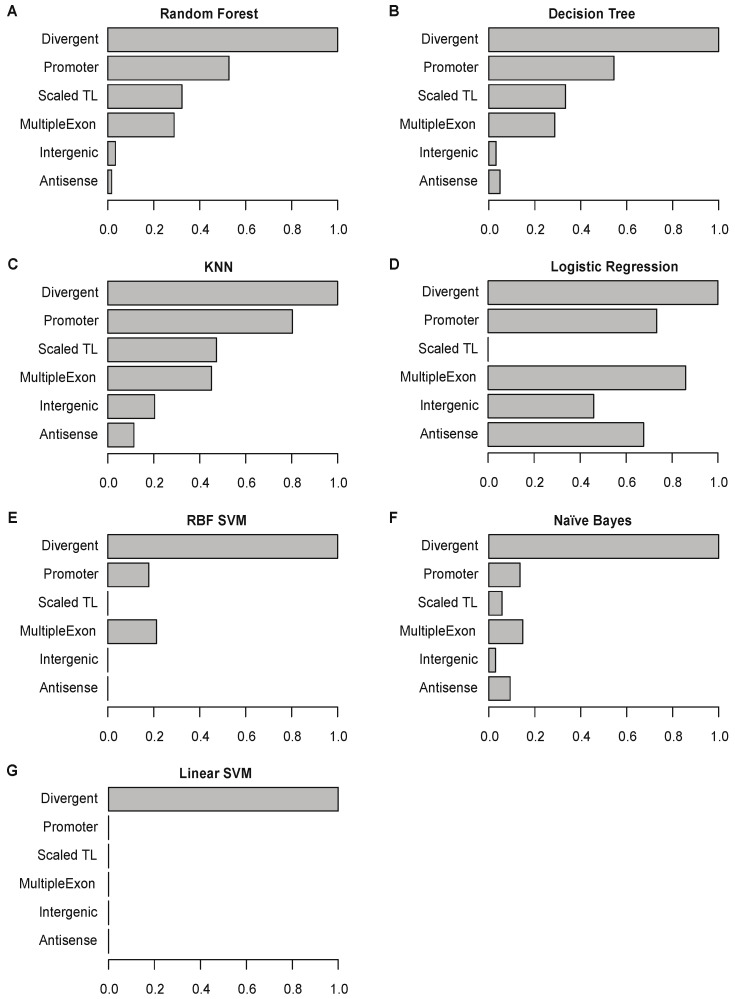
Feature importance for each of the seven final models. Each graph shows the scaled importance scores for each feature for the given model: random forest (**A**), decision tree (**B**), KNN (**C**), logistic regression (**D**), support vector machine (SVM) with RBF kernel (**E**), Naïve Bayes (**F**), and SVM model with linear kernel (**G**) models. For each model, the importance scores of all features were scaled to (0.1) by dividing the original score of the most significant feature (see Section 4).

We evaluated the significance of the selected genomic features for each ML model. The feature importance score indicates the relative importance of each genomic feature (transcript length, promoter signature, multiple exons, and genomic location) for Flnc in detecting true lncRNAs from RNA-seq data. Because the genomic location feature includes three categories (divergent, antisense, and intergenic), we evaluated the feature importance for six categories: transcript length, promoter signature, multiple exons, divergent transcript, antisense transcript, and intergenic transcript. We found that divergent transcript feature is the most important category across all ML models (Figure 6). For three models—random forest, decision tree, and KNN—promoter signature ranks as the second most important feature. In contrast, the intergenic and antisense transcript features are the least important in all models (Figure 6).

### 2.8. Flnc Achieves Similar Performance at the lncRNA Gene Locus Level as at the Transcript Level

More than half (57%) of the true lncRNAs in the benchmark dataset have multiple exons. Among them, 43% of multi-exon lncRNA genes have undergone alternative splicing events, generating multiple lncRNA isoforms from the same lncRNA gene locus. *Flnc* has been trained and tested at the transcript level for each RNA-seq dataset. To examine the performance of *Flnc* at the locus level, we further tested *Flnc* on the lncRNA gene loci which encode only lncRNA transcripts. This test showed that *Flnc* can achieve similar performance at the lncRNA gene locus level as at the lncRNA transcript level (Supplementary Figure S5), although *Flnc* performs better at the transcript level than at the locus level.

## 3. Conclusions and Discussion

We have developed a comprehensive pipeline and software *Flnc*, which uses ML models to accurately identify true lncRNAs from RNA-seq data. *Flnc* does not require matched transcription initiation profiles which are usually marked by H3K4me3 histone modifications. We trained the ML models on four types of features, transcript length, promoter signature, multiple exons, and genomic location, which show a high degree of discrepancy between true lncRNAs and false lncRNAs in our benchmark datasets. We benchmarked *Flnc* on both the transcript level, using all 46 datasets, and the dataset level, using the 46 RNA-seq datasets individually. *Flnc* can achieve up to 85% prediction accuracy using only RNA-seq data. This software improves of prediction of true lncRNAs from samples, such as clinical samples, where ChIP-seq data are unavailable. *Flnc* can save time and resources by making the generation of H3K4me3 unnecessary and allowing the identification of lncRNAs from publicly available RNA-seq data.

The predictive power of *Flnc* relies on the quality of the benchmark datasets and the identification of true and false lncRNA within them. Therefore, the prediction power of *Flnc* may be limited by factors that affect the reliability of the benchmark datasets. These factors include RNA-seq and ChIP-seq data quality, accuracy of transcript assembly and ChIP-seq peak calling, and sequencing depth. To minimize the effects of these factors, we examined the data quality and sequencing depths of both RNA-seq and ChIP-seq data in the benchmark datasets and only keep the data with good data quality and adequate sequencing depths in this study. To improve the accuracy of transcript assembly, we integrated multiple transcript-assembly methods. It has been shown that the accuracy of ChIP-seq peak calling can be improved by using different approaches for long (≥70 bp) and short (<70 bp) reads studies [44,45], and we, therefore, used this approach.

Compared to the true lncRNAs identified by H3K4me3 profiles, true lncRNAs predicted by *Flnc* (with random forest) are more likely to be divergent transcripts of nearby protein-coding genes and to have promoter signatures (Supplementary Figure S4B). This result suggests that *Flnc* might overestimate divergent putative lncRNAs and putative lncRNAs with promoter signatures as true lncRNAs, but underestimate the true lncRNAs that are in intergenic regions. One possible explanation for this is that divergent transcription and promoter signature are the two most important features for the three best models (random forest, decision tree, and KNN).

Because different methods of generating and sequencing RNA-seq libraries result in slightly different collections of transcripts, feature importance may determine model performance for different types of RNA-seq data. We found that the random forest, decision tree, and KNN models outperform than other models for stranded RNA-seq data generated from polyA-selected RNA (Figure 3 and Supplementary Figure S3). In contrast, the linear SVM and naïve Bayes models, which performed poorly for stranded polyA-selected RNA-seq data, achieve better prediction accuracy and precision than other models for rRNA-depleted and unstranded RNA-seq data. This may be because the random forest, decision tree, and KNN models rely on three major features: divergent transcript, promoter signature, and transcript length (Figure 6), but the transcript length feature may not be applicable to nascent lncRNAs, which are included in rRNA-depleted RNA-seq data. In contrast, for unstranded RNA-seq data, the promoter signature feature may not applicable because without accurate strand information, the promoters of these putative lncRNA cannot be inferred. Unlike the random forest, decision tree, and KNN models, the linear SVM and naïve Bayes models mainly rely on the divergent genomic structure feature (Figure 6), which is applicable for both nascent and unstranded lncRNAs.

*Flnc* includes seven embedded ML models (random forest, decision tree, logistic regression, naïve Bayes, KNN, linear-SVM, and RBF-SVM) and an ensemble approach, which makes *Flnc* suitable for identifying lncRNAs from different types of RNA-seq data. Model performance depends somewhat on the type of RNA-seq data that is used as input. The best three models (random forest, decision tree, and KNN) for the stranded polyA-selected RNA-seq data achieve very high consistency (93–96%) in true lncRNA prediction (Supplementary Figure S3D). Therefore, we recommend that users select any of these three models when using *Flnc* to identify lncRNAs from stranded polyA-selected RNA-seq data. Precision can be further improved by the ensemble approach (Supplementary Figure S3E). Therefore, we recommend using the *ensemble* setting of the *Flnc* software, which provides users with the common set of true lncRNAs predicted by all models.

Although *Flnc* was trained on the benchmark datasets of lncRNAs identified from stranded polyA-selected RNA-seq data and matched H3K4me3 ChIP-seq data, the *Flnc* pipeline can be applied to identify lncRNAs from other types of RNA-seq data, such as stranded rRNA-depleted RNA-seq data and unstranded polyA-selected data. Based on the performance of the seven models on stranded rRNA-depleted RNA-seq data and unstranded polyA-selected data, we recommend using the linear KNN or naïve Bayes models for these two types of RNA-seq data.

The *Flnc* software can identify novel lncRNAs directly from RNA-seq data, as well as evaluate whether a transcript is a lncRNA. *Flnc* can take three types of input files, including raw RNA-seq data in the FASTQ format, transcript coordinates in the BED format, and transcript sequences in the FASTA format. For maximum portability and usability, *Flnc* is implemented in in Python 2 and the entire pipeline is wrapped within a Singularity container. The current version of *Flnc* is applicable to data of human samples, but the concept level of *Flnc* is applicable to other organisms. As a follow-up study, we plan to extend *Flnc*’s method to be applicable to other organisms.

## 4. Material and Methods

### 4.1. Collection of Sequencing Datasets to Generate Benchmark lncRNAs

Because most mature lncRNAs have polyA tails [2] and clear transcriptional start sites, generating a benchmark dataset of true and false lncRNAs requires stranded polyA-selected RNA-seq and sample-matched H3K4me3 ChIP-seq. We identified 388 data series with the keywords “RNA seq,” “H3K4me3,” and “*Homo sapiens*” from the NCBI GEO database through February of 2021. From the 388 GEO studies, we selected 61 datasets with available sample-matched, stranded polyA-selected RNA-seq and H3K4me3 ChIP-seq data.

For the 61 datasets, we examined the quality and sequencing depths of RNA-seq and ChIP-seq data and removed 15 datasets, which had poor RNA-seq or ChIP-seq data quality or extremely low sequencing depths. We considered the quality of RNA-seq data poor if more than half of the reads had quality control scores less than 35. For ChIP-seq data, we considered their quality poor if the number of called peaks in the dataset was an outlier among peak numbers found in the 61 datasets (extremely small < 2000 or extremely large > 200,000). This process resulted in selection of 46 datasets. The RNA-seq and ChIP-seq data within each of the selected 46 high-quality datasets were generated from the same type of sample. The datasets are heterogenous, including both single-end (50%) and paired-end (50%) RNA-seq data and each dataset has one to four RNA-seq replicates. We indexed these datasets 1 to 46 in chronological order by submission date (Supplementary Tables S1–S3) and used them to identify true and false lncRNAs in each dataset as benchmark data.

After identifying true and false lncRNAs in each dataset (see below), we split the benchmark dataset into training and testing sets based on the submission dates of the RNA-seq data into the NCBI GEO database [46]. The training set included the 41 datasets that were submitted to the GEO database before 2019; we used the five datasets generated in 2019 and 2020 as the testing set.

### 4.2. Identification of Putative lncRNAs

First, we mapped the RNA-seq data to the human reference genome (hg38) using HISAT2 v 2.0.5 [47] and assembled transcripts using both StringTie v1.3.4 [22] and Strawberry v1.1.2 [23]. For each sample, we then merged assembled transcripts of replicates with the StringTie merge function. Next, we examined the coding potential of the assembled transcripts using CPAT v1.2.4 [25], LGC v1.0 [26], PLEK v1.2. [27], and CPPred [28]. We excluded the transcripts with coding potential defined in any of the above tools. Next, we excluded the resulting transcripts, as well as any transcripts that overlapped protein-coding genes or pseudogenes on the same strand. We also excluded transcripts overlapping other annotated noncoding RNAs, including snoRNA, rRNA, tRNA, and microRNAs, on the same strand. From the remaining transcripts, we selected expressed transcripts that were over 200 nucleotides long as putative lncRNAs (see Supplemental Methods for details).

### 4.3. Identification of H3K4me3 Peaks Using H3K4me3 ChIP-Seq Data

We aligned H3K4me3 ChIP-seq reads to the human reference genome (hg38/GRCh38) using the BWA v0.7.5a toolkit [44]. For reads with mean read length ≥ 70 bp we used BWA-MEM and for short reads we used BWA-aln. Next, we used the MACS2 v2.2.7.1 [48] peakcall function to identify H3K4me3, as described previously [18], with the following settings: -q 0.01 --broad --broad-cutoff=0.01 --nomodel --extsize 300. For H3K4me3 ChIP-seq data without matched control data, MACS2 called peaks based on the H3K4me3 input ChIP-seq data. For H3K4me3 ChIP-seq data with matched control data, MACS2 called peaks by comparing the bam files to the matched background control. We used the H3K4me3 broad peaks called by MACS2 as transcription initiation markers [49,50].

### 4.4. Identification of True and False lncRNAs Based on H3K4me3 ChIP-Seq Data

We used our previously established approach [18] to identify true and false lncRNAs among putative lncRNAs. Because H3K4me3 chromatin modification is well known as transcriptional initiation marks of active genes [34], including lncRNA genes, we examined the distance between the 5′ ends of putative lncRNAs and the matched H3K4me3 peaks. If the H3K4me3 peak was within 1 kb of the 5′ end of a putative lncRNA, we considered it a true lncRNA; otherwise, we considered it false.

### 4.5. Normalization of Transcript Lengths

We calculated the length of putative lncRNAs by summing the length of all the transcript’s exons. Next, we log-transformed the lengths. We calculated the upper and lower limits as mean plus and minus 3 × standard deviation, respectively. Then, we set the outlier data points of the log-transformed lengths as the value of upper or lower limits. Finally, we scaled the log-transformed values of transcript lengths for each putative lncRNA into the range between 0 and 1 using the min-max normalization technique.

### 4.6. Identification of Promoter Regions

We used TSSG software [41] to identify the potential promoters in the genomic regions±1 kb of the 5′ end of putative lncRNAs. TSSG detects promoters by scanning for transcription factor binding sites and is considered to be one of the most accurate mammalian promoter prediction programs with the fewest false positive predictions [51].

### 4.7. Classification of Putative lncRNAs by Genomic Location

We used the genomic locations of protein-coding genes annotated by the GENCODE Project release 29 [52], and compared them to the genomic locations of putative lncRNAs using the BEDTools suite [53] and GffCompare tool [54]. We classified putative lncRNAs—true and false—into three categories by genomic locations [18,35]: divergent, antisense, and intergenic. A putative lncRNA was classified as divergent if the 5′ end of its locus was within ±2 kb of the TSS of a protein-coding gene on the opposite strand [18,35]. The remaining putative lncRNAs that were antisense to protein-coding genes and overlapped the gene by at least one base pair were classified as antisense. All remaining putative lncRNAs were classified as intergenic.

### 4.8. Calculation of the Exon Numbers of Putative lncRNAs

The number of exons of putative lncRNAs were counted based on the assembled transcripts in GTF format, which include the information of exon numbers and coordinates of each exon.

### 4.9. Measurement Feature Importance

To determine the feature importance for all machine learning models, we used the permutation approach [55]. We scaled the importance scores into the range between 0 and 1 by dividing each importance score by the importance score of the most significant feature.

### 4.10. Collection of Other RNA-Seq Datasets and Matched H3K4me3 ChIP-Seq Data

To evaluate *Flnc*’s performance on other types of RNA-seq data, we collected four additional datasets, each corresponding to one sample from the GEO database (Supplementary Table S4). These four datasets all contained RNA-seq data and matched H3K4me3 ChIP-seq. Two datasets included stranded rRNA-depleted RNA-seq (accession numbers: GSE179184 and GSE189861) and two included unstranded polyA-selected RNA-seq data (accession numbers: GSE72131 and GSE58740).

## Data Availability

The datasets analyzed in this study are available in GEO with accession numbers listed in Supplementary Table S1. These datasets were downloaded from the NCBI GEO database: https://www.ncbi.nlm.nih.gov/geo/, accessed on 10 September 2022. The constructed benchmark dataset of true and false lncRNAs are available at https://zhoulab.umassmed.edu/Flnc_data/, accessd on 10 September 2022. The analyses were performed with *Flnc* version 1. The *Flnc* software is freely available on GitHub: https://github.com/CZhouLab/Flnc, accessed on 10 September 2022 along with a tutorial.

## Supplementary Materials

The following supporting information can be downloaded at: https://www.mdpi.com/article/10.3390/ncrna8050070/s1. Figure S1: True and false lncRNAs in each of the 46 benchmark datasets. Figure S2: Genomic features of true and false lncRNAs in each of the 46 benchmark datasets. Figure S3: Performance of the seven final ML models used in *Flnc*. Figure S4: The genomic features of true lncRNAs predicted by *Flnc* and true lncRNAs determined by H3K4me3 profiles in the test set. Figure S5: *Flnc* achieves similar performance at the lncRNA gene locus level as at the transcript level. Table S1: Information of the collected 46 datasets, each of which includes both of stranded polyA-selected RNA-seq data and matched H3K4me3 ChIP-seq data from the same human sample. Table S2: The mapping information for the RNA-seq data and ChIP-seq data among the 46 datasets used in this study. Table S3: Performance of the conventional approach and the seven ML models of *Flnc* in the training set and test datasets. Table S4: Information of four datasets with other types of RNA-seq data.
