# Supplementary material for: Flnc: Machine Learning Improves the Identification of Novel Long Noncoding RNAs from Stand-Alone RNA-Seq Data
Source: Noncoding RNA. 2022 Oct 13;8(5):70. doi: 10.3390/ncrna8050070 (PMC9607125; doi:10.3390/ncrna8050070)
Supplement: Supplementary file 1 [file ncrna-08-00070-s001.zip › Suppl_Methods_And_SFigs_legends.pdf]

**SUPPLEMENTAL DATA FOR THE PAPER "*Flnc*: Machine Learning Improves the Identification of Novel Long Noncoding RNAs from Stand-Alone RNA-Seq Data**"

## SUPPLEMENTAL METHODS

**Identification of putative lncRNAs**

We upgraded the previous computational pipeline (**Fig 1A**) [18,35] with the latest tools to identify putative lncRNAs from raw RNA-seq data. The putative lncRNAs are transcripts longer than 200nt that lack coding ability. To identify the putative lncRNAs, we first assembled transcripts from raw RNA-seq data, then filtered out the transcripts that fall into any of the following categories: (i) transcripts with potential coding ability or other small noncoding RNAs, (ii) transcripts shorten than 200 nucleotide or with extremely low expression levels. Please see the details as follows.

**1) *Ab initio* assembly of transcripts from RNA-seq data**

We mapped each replicate of stranded polyA-selected RNA-seq data to the human reference genome (hg38/GRCh38) using HISAT2 v2.0.5 [47], and then assembled transcripts using StringTie v1.3.4 [22] and Strawberry v1.1.2 [23].

The HISAT2 settings for single-end RNA-seq data were as follows:
*hisat2 -p 10 --dta -x < index of reference genome> -U < Reads.fastq > --add-chrname --rna-strandness <strandness> --fr --known-splicesite-infile <known splice site> --novel-splicesite-outfile <novel splice site file> --novel-splicesite-infile <novel splice site file> --seed 168 --phred33 --min-intronlen 20 --max-intronlen 500000*

The HISAT2 settings for paired-end RNA-seq data were as follows:
*hisat2 -p 10 --dta -x <index of reference genome> -1 <Reads_end1.fastq> -2 <Reads_end2.fastq> --add-chrname --rna-strandness <strandness> --fr --known-splicesite-infile <known splice site> --novel-splicesite-outfile <novel splice site file> --novel-splicesite-infile <novel splice site file> --seed 168 --phred33 --min-intronlen 20 --max-intronlen 500000*

The reference genes in GTF file format (genes.gtf) were downloaded from the Release 29 version of the GENCODE database [52]. We then assembled transcripts with the following settings of StringTie and Strawberry using HISAT2's output bam file as input:

*stringtie <strandness> -p 20 -G genes.gtf -o d <output.gtf> -l ${1} -f 0 -m 200 -a 10 -j 1 -M 1 -g 50 <HISAT2_output_bam_file>*

*strawberry <strandness> -g genes.gtf -o <output.gtf> -p 20 -m 0 -t 200 -s 10 -d 50 --no-quant --min-depth-4-transcript 0.1 <HISAT2_output_bam_file>*

Next, all transcripts assembled by either StringTie or Strawberry from all replicates were merged into one list through the StringTie merge function.

**2) Remove transcripts with potential coding abilities or other small noncoding RNAs.**

First, we removed transcripts that overlapped with annotated protein-coding genes, pseudogenes, rRNAs, tRNAs, small nucleolar RNAs (snoRNAs), and microRNAs on the same strand. Second, we removed transcripts with protein coding potential. The coding potential of each remaining transcript was estimated by CPAT v1.2.4 [25], LGC v1.0 [26], PLEK v1.2 [27], and CPPred [28]. We used the default threshold or the suggested threshold of each tool to determine the

coding abilities for each of the remaining transcripts. Third, we removed any remaining transcripts that overlapped on the same strand with the transcripts removed in the previous two steps.

3) **Remove short and lowly expressed transcripts**
We removed the remaining transcripts that were shorter than 200nt. Then, we removed the remaining transcripts that are lowly expressed. A transcript is considered lowly expressed if it had either 1) less than ten reads per transcript, or 2) its expressed level is less than the smallest FPKM peak value that occurs in the bottom half of the FPKM distribution curve for all remaining expressed transcripts.

## SUPPLEMENTAL FIGURES

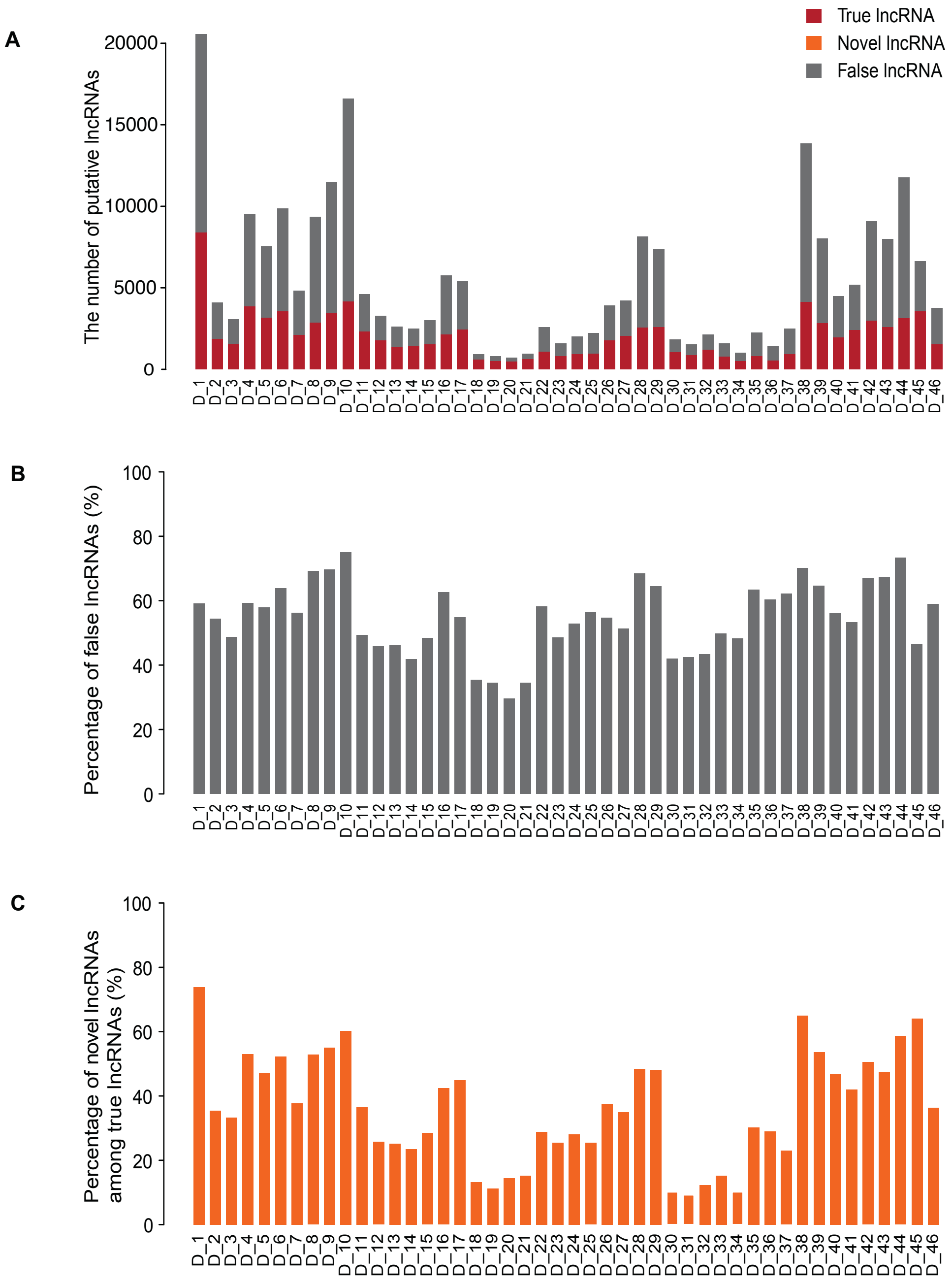


Supplemental Figure S1: True and false lncRNAs in each of the 46 benchmark datasets. The number of putative true (red) and false (grey) lncRNAs (A) and the percentage of false lncRNAs in each dataset (B). The putative true lncRNAs include a high percentage of novel lncRNAs in each dataset (C).

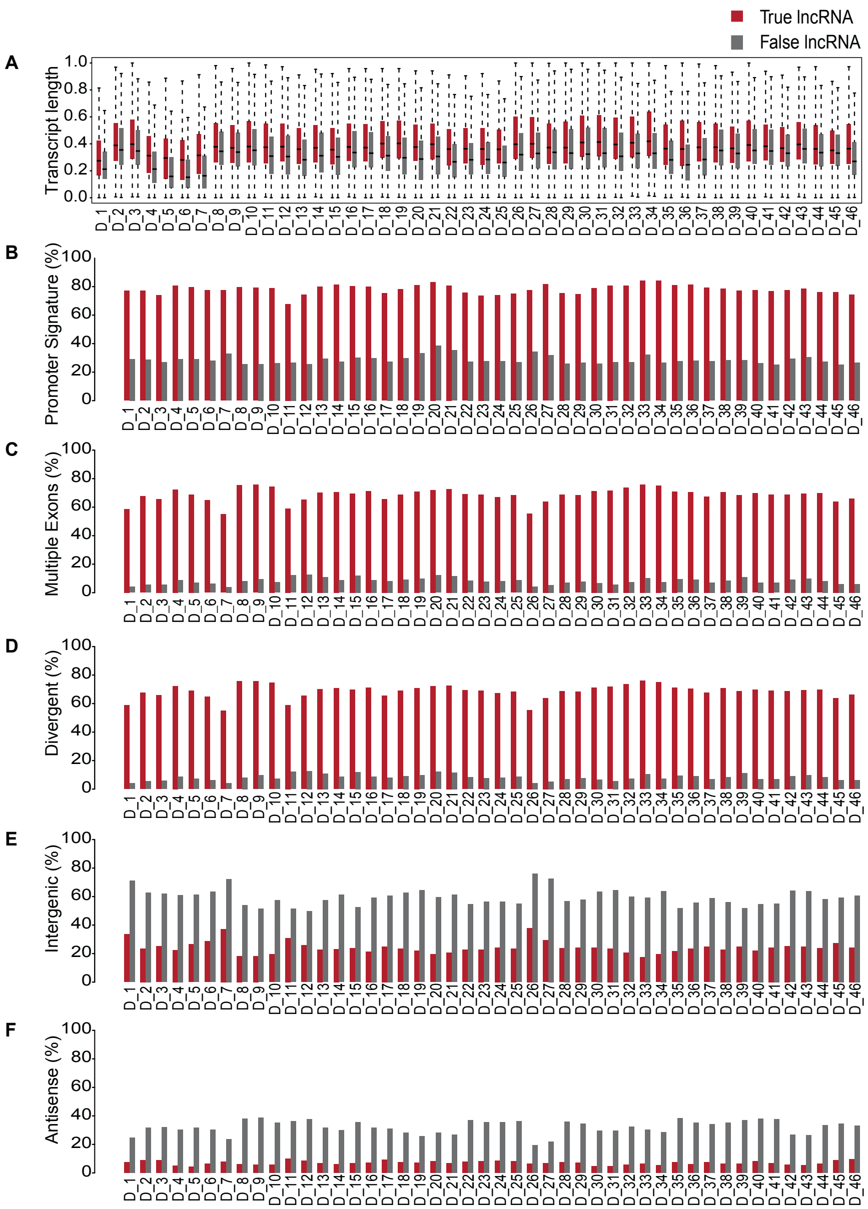


Supplemental Figure S2: Genomic features of true and false lncRNAs in each of the 46 benchmark datasets. In each of the 46 benchmark datasets, true and false lncRNAs can be distinguished by (A) transcript length (A), the presence of an upstream promoter signature (B), the presence of multiple exons (C). True lncRNAs are more often divergently transcribed from the promoters of protein-coding genes (D), and less likely to be intergenic (E), or antisense to protein-coding genes (F).

*For (A), the boxplots represent the scaled transcript lengths of true lncRNAs and false lncRNAs across each of the 46 benchmark datasets. The error bars are the 95% confidence interval, the bottom and top of the box are the 25th and 75th percentiles, the line inside the box is the 50th percentile (median). For (B-F), each bar-plot represents the percentage of a feature among the true and false lncRNAs across each of the 46 benchmark datasets.*

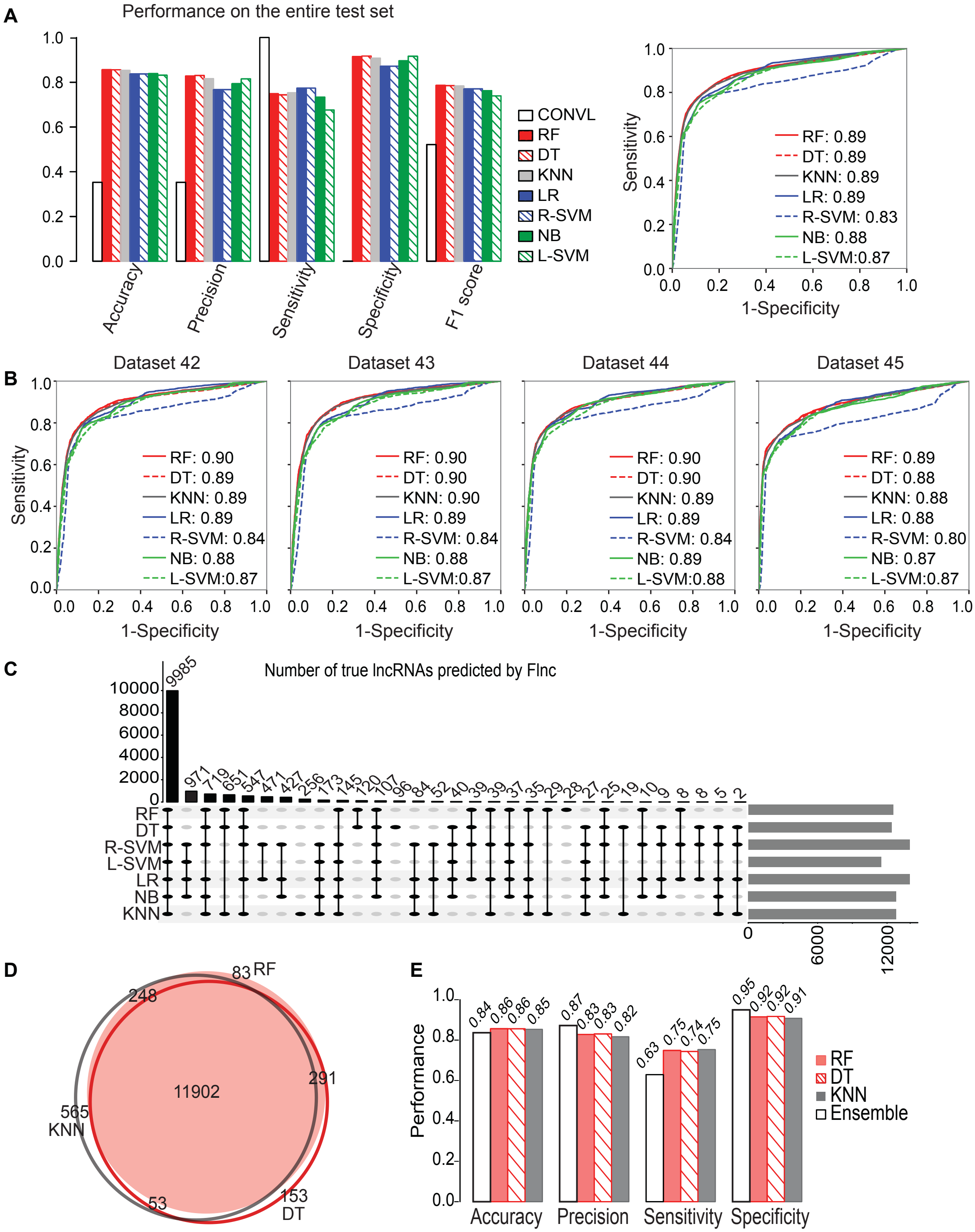
A
Performance on the entire test set
Accuracy
Precision
Sensitivity
Specificity
F1 score
CONVL
RF
DT
KNN
LR
R-SVM
NB
L-SVM
Sensitivity
1-Specificity
RF: 0.89
DT: 0.89
KNN: 0.89
LR: 0.89
R-SVM: 0.83
NB: 0.88
L-SVM:0.87
B
Dataset 42
Dataset 43
Dataset 44
Dataset 45
C
Number of true lncRNAs predicted by Flnc
D
11902
E
Performance
Ensemble

Supplemental Figure S3: Performance of the seven final machine learning models used in *Flnc*. (A) The seven final models of *Flnc* outperformed the conventional method (without models) on the entire test set. *The left bar graph shows the performance metrics accuracy, precision, sensitivity, specificity and F1 score. The right graph shows the receiver operating characteristic (ROC) curve for each model. Area Under the ROC curve (AUROC) score is shown next to each ML model.* (B) The ROC curves on the dataset 42-45 within the test set. (C) The upset plot shows the number of true lncRNAs commonly predicted by different models. The vertical bars in black represent the number of true lncRNAs commonly predicted by the models that are highlighted and connected by black line below the bar. For example, 9985 true lncRNAs are commonly predicted by all the seven models in the test set; and 971 true lncRNAs are commonly predicted by RBF SVM, linear SVM, logistic regression and naïve Bayes models. The horizontal bars in gray represent the number of true lncRNAs predicted by each model listed on the left side. (D) The Venn diagram of the predicted true lncRNAs by the three best models (random forest, decision tree and KNN). (E) Comparison of the performance of the three best models and the ensemble approach (in white). The result predicted by ensemble approach will improve the prediction precision and specificity with the cost of a reduced sensitivity.
*Abbreviations: CONVL, conventional approach; RF, random forest; DT, decision tree; R-SVM, RBF support vector machine; L-SVM, linear support vector machine; LR: linear regression; NB, naïve bayes; KNN,* k-nearest neighbors.

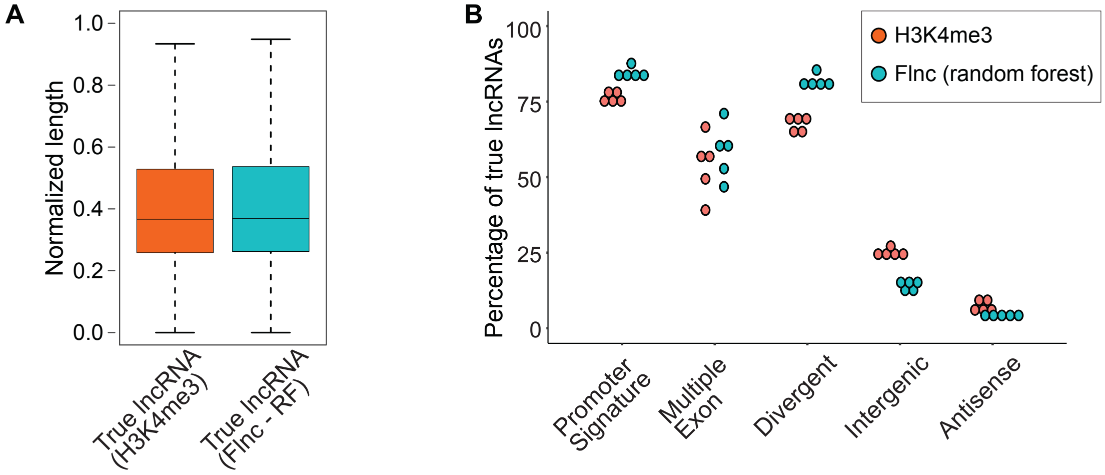


Supplemental Figure S4: The genomic features of true lncRNAs predicted by *Flnc* and true lncRNAs determined by H3K4me3 profiles in the test set. (A) The true lncRNAs predicted by *Flnc* (with random forest model) show similar normalized transcript length distribution as the true lncRNAs determined by H3K4me3 ChIP-seq data. (B) The true lncRNAs predicted by *Flnc* (with random forest model) include significantly more divergent transcripts and transcripts with promoter signatures than the true lncRNAs determined by H3K4me3 ChIP-seq data, whereas multiple exon features exhibit similar percentage between these two groups of lncRNAs.

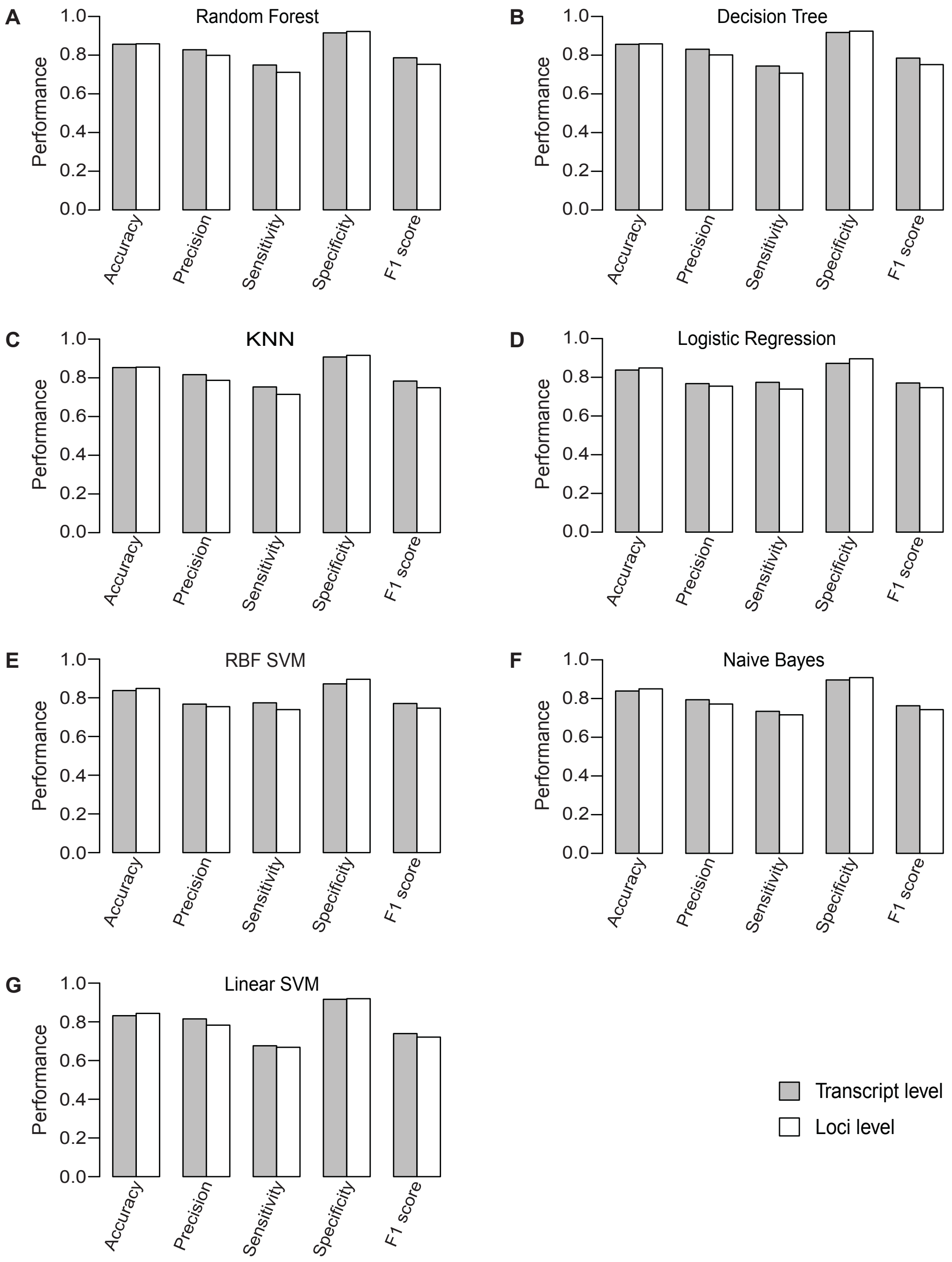


Supplemental Figure S5: *Flnc* achieves similar performance at the lncRNA gene locus level as at the transcript level. Each graph shows the performance for the given model: random forest (A), decision tree (B), KNN (C), logistic regression (D), support vector machine (SVM) with RBF kernel (E), naïve bayes (F), and SVM model with linear kernel (G) models.
